# NORAD regulates epithelial-mesenchymal transition of non-small cell lung cancer cells via miR-422a

**DOI:** 10.3892/mmr.2021.12178

**Published:** 2021-05-27

**Authors:** Zhikun Chen, Qin Che, Chunxue Xie

Mol Med Rep 23: Article no. 111, 2021; DOI: 10.3892/mmr.2020.11750

Following the publication of this paper, the authors have realized that they overlooked indicating that Zhikun Chen and Qin Che contributed equally to this work. Therefore, the affiliations for this paper should have been written as follows:

ZHIKUN CHEN^1*^, QIN CHE^2*^ and CHUNXUE XIE^3^

Departments of ^1^Emergency, ^2^Infectious Diseases and ^3^General Practice, Jingmen No. 1 People's Hospital, Jingmen, Hubei 448000, P.R. China

*Contributed equally

The authors confirm that there are no further errors in the study, and all the authors agree to this correction. The authors regret their oversight, and apologize for any inconvenience caused.

